# Sexual activity increases resistance against *Pseudomonas entomophila* in male *Drosophila melanogaster*

**DOI:** 10.1186/1471-2148-13-185

**Published:** 2013-09-06

**Authors:** Vanika Gupta, Zeeshan S Ali, Nagaraj G Prasad

**Affiliations:** 1 Indian Institute of Science Education and Research, Mohali, India

**Keywords:** Trade-offs, *P. entomophila*, *S. succinus*, Resistance

## Abstract

**Background:**

Maintenance and deployment cost of immunity is high, therefore, it is expected to trade-off with other high cost traits like sexual activity. Previous studies with *Drosophila melanogaster* show that male’s ability to clear bacteria decreases with increase in sexual activity. We subjected this idea to test using two pathogens (*Pseudomonas entomophila* and *Staphylococcus succinus*) and three different populations of *Drosophila melanogaster*.

**Results:**

We found that sexual activity enhanced male survivorship in a pathogen specific manner. Sexually active males show higher resistance than virgins upon infection with *Pseudomonas entomophila*. Interestingly, the beneficial effects of sexual activity increased with time of co-habitation with females and declined when access to females was restricted. We observed no change in male survivorship upon experimentally varying the number of sexual interactions.

**Conclusion:**

Our results show that the sexual activity-immunity trade-off in males cannot be generalised. The trade-off is potentially mediated through complex interactions between the host, pathogen and the environment experienced by the host.

## Background

According to life history theory, if the maintenance of two traits is energetically costly, they are very likely to trade-off with each other [1,2]. In promiscuous species like *Drosophila melanogaster*, males are selected to invest a substantial amount of resources in pre-copulatory traits such as courtship and post-copulatory traits such as sperm competition. At the same time, such organisms also have to maintain an energetically costly immune system in order to fight against a plethora of pathogens. Thus among males, sexual activity and immunity are likely to trade-off with each other [3,4].

Multiple studies have tried to address the proposed trade-off between immunity and sexual activity. In one set of studies, increasing the level of sexual activity decreased components of immunity like phenoloxidase activity, encapsulation, hemocyte load, hemolytic activity and melanization in males of different insects [5-8]. Another set of studies have found that infection extracts a cost in terms of reduced ejaculate quality and secondary sexual characters in males [9,10]. Sperm viability shows a negative genetic correlation with humoral components of immunity and a positive genetic correlation with cellular components of immunity [11]. However, in Australian crickets, increased number of matings did not change lytic activity (an important component of immune system) [12]. Singly mated males of *Tenebrio molitor* had better resistance to fungal infections compared to virgin males [13]. Thus, the results about immunity- sexual activity trade-off in males have been fairly variable across studies. It is quite likely that at least a part of this variability is due to the different host-pathogen systems used and the components of immunity assayed across studies.

Phenotypic and experimental evolution studies in *Drosophila melanogaster*[5,14,15] suggest that under conditions of increased sexual activity, males show lower ability to clear bacteria from their body - a result interpreted to be in accordance with the proposed trade-off between sexual activity and immunity. Most of the previous studies in *Drosophila* addressing this issue [5,14,15] have used a bacterial strain (*E. coli*) which does not kill wild type flies but is pathogenic to flies which are immunodeficient. Even though *E.coli* has been successfully used as a model organism for the study of immunity, it is important to look at immunity and associated trade-offs using other pathogens which are capable of growing within a wild-type host and hence can establish a sustained infection. The present study addresses the effect of sexual activity on the post infection survivorship of *Drosophila melanogaster* males when they were challenged by two such pathogens: *Pseudomonas entomophila* and *Staphylococcus succinus* isolated from wild caught flies. We also measured bacterial load 24 hours post infection whenever a change in survivorship post infection was observed in order to tease out whether changes in *resistance* or in *tolerance* was responsible for change in post infection survivorship. ‘Resistance’ is defined as the ability to limit parasite burden and ‘tolerance’ as ability to limit damage caused by given parasite burden [16].

For our study, we used three different types of host systems -a recently wild-caught population (BRB) which has been maintained under laboratory conditions for about 20 generations, a long term laboratory adapted population (LH) which has been maintained under laboratory conditions for more than 300 generations and the standard laboratory line Canton S- and two pathogens -*Pseudomonas entomophila* (Pe) and *Staphylococcus succinus* (Ss). Pe is a highly virulent pathogen (causes about 70% mortality) isolated from *Drosophila melanogaster*[17]. Ss is a weak pathogen (causes about 40% mortality) recently isolated from wild- caught fruit flies in Chandigarh, India (Singh and Prasad unpublished data). Our results do not conform to the idea of a trade-off between reproduction and immunity. Males’ survivorship when infected with one of the two pathogens used in this study (viz. Pe) was found to improve with reproductive activity, with synergistic effects on males’ resistance to this pathogen. We argue that our results challenge the generality of the idea of immunity-sexual activity trade-off and propose that such trade-off might be highly system specific.

## Results

### Sexual activity enhances survivorship when infected with Pe

**Figure 1:**
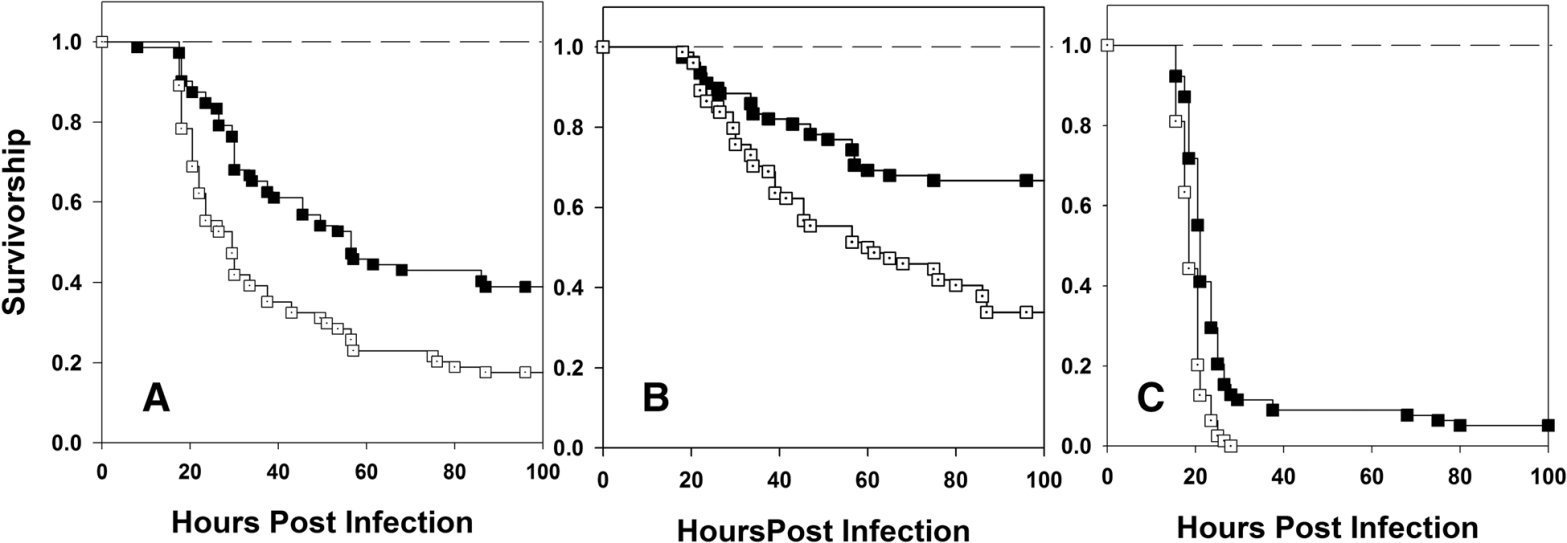
**Effect of sexual activity on male survivorship after infection with *P. entomophila.*** Kaplan-Meier plots of survivorship of sexually active (■) and virgin males (□) from BRB **(A)**, LH **(B)** and CS **(C)** populations. The experiment was done in two independent blocks and 40 males were infected in each Block × Treatment × Population combination. Data was analysed using two models- model1: that includes block as random factor (using R package ‘coxme’ [25]) and model2: that does not include block as random factor (using R package ‘coxphw’ [26]; for details see statistical analysis section in materials and methods). No significant block effects were detected and thus block was eliminated as a factor and data from the two blocks were pooled for further analysis. When infected with *P. entomophila*, sexually active males survived significantly better than virgin males in all the populations (all p < 0.0001, Cox proportion regression analysis). None of the sham infected flies (―――) died over the course of the observation. While we did have mated and virgin sham infected controls, we represent both of them with a single line in the figure for simplicity.

**Figure 2:**
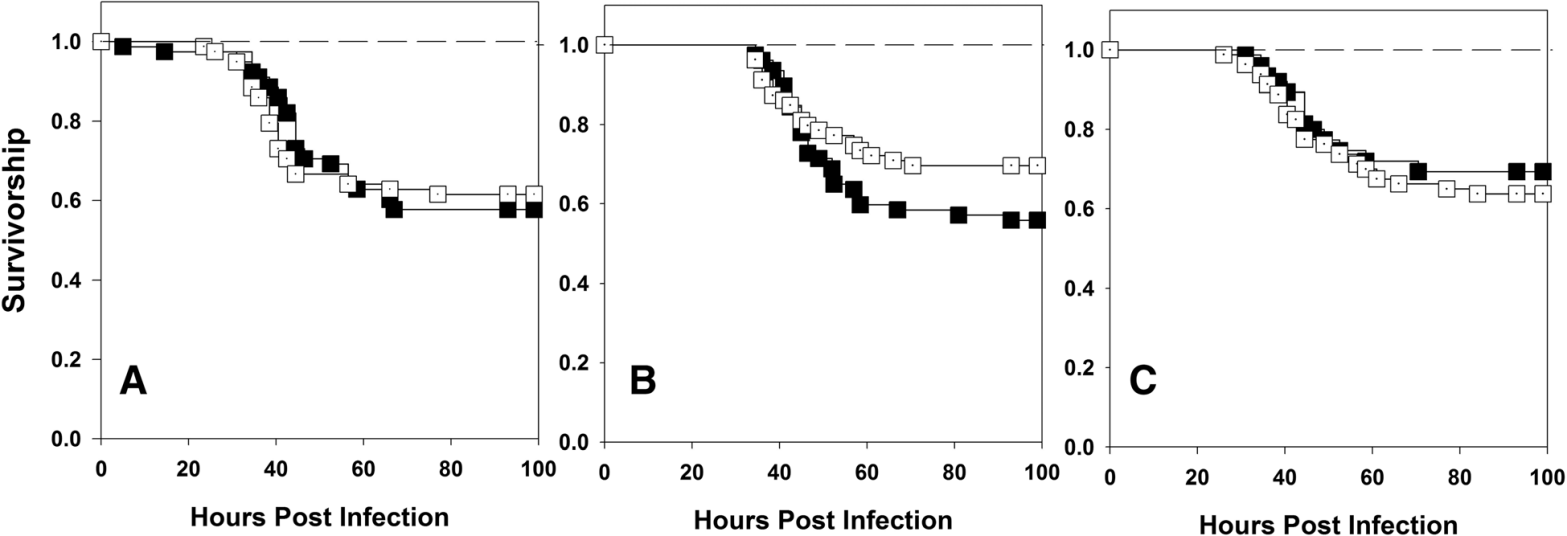
**Effect of sexual activity on male survivorship after infection with *S.succinus.*** Kaplan – Meier plots of survivorship of sexually active (■) and virgin males (□) of populations BRB **(A)**, LH **(B)** and CS **(C)**. The experiment was done in two independent blocks and 40 males were infected in each Block × Treatment × Population combination. Data was analyzed using two models- model1: that includes block as random factor (using R package ‘coxme’ [25]) and model2: that does not include block as random factor (using R package ‘coxphw’ [26]; for details see statistical analysis section in materials and methods). No significant block effects were detected and thus block was eliminated as a factor and data from the two blocks were pooled for further analysis. When infected with *S.succinus,* no difference in survivorship of sexually active and virgin males was observed (Panel **A**, *p* = 0.99; Panel **B**, *p* = 0.14 and Panel **C**, *p* = 0.31, Cox proportion regression analysis). None of the sham infected flies (―――) died over the course of the observation. While we did have mated and virgin sham infected controls, we represent both of them with a single line in the figure for simplicity.

Virgin males were collected on 10th day post egg collection. It was ensured that the flies were virgins by collecting them within 6 hours of eclosion (flies in our system start mating only 8 hours post eclosion). On 12th day post egg collection, the males were randomly assigned to one of the two treatments- Sexually active or virgin. Males from the sexually active treatment were combined with females and allowed to interact for two days in fresh food vials. Males from the virgin treatment were transferred to fresh food vials without any females. On 14th day post egg collection, males from the virgin and sexually active treatments were infected by pricking with a fine needle dipped in bacterial culture (10 mM MgSO_4_ for both virgin and mated controls). Numbers of dead individuals were recorded at regular intervals (4 hours for initial 24–60 hours and then 6–8 hours for 60–100 hours post infection). We found that virgin males had lower survivorship and higher mortality rate compared to sexually active males when flies were challenged with Pe (Figure 1). The data was modeled in two ways- (a) model 1: that includes block as a random factor (using R package ‘coxme’ [18]) and (b) model 2: that does not include block as a random factor (using R package ‘coxphw’ [19]). Analysis of deviance was performed comparing the log-likelihood ratio estimates of the two models. The log-likelihood ratios were not found to be different in any of the cases, thereby eliminating an effect of block (Table 1). Survivorship analysis using Cox proportion regression analysis model and Kaplan - Meier estimator indicated significant differences among virgin and mated males (Table 2). This result was consistent across the three host populations (Table 2). However, when flies were challenged with Ss, we found no significant difference between mated and virgin males in their survivorship or mortality rate (Figure 2). This result was again consistent across host populations (Table 2). None of the sham infected controls died.

**Table 1:** Summary of results from analysis of deviance between two models using log- likelihood ratio estimates to analyze the block differences (model1. ~treatment + (1 + treatment | block); model2. ~treatment)

**Pathogen**	**Population**	**Log-likelihood ratio**	**χ**^**2**^	**DF**	**p > | χ |**
***Pseudomonas entomophila***	BRB	model1. -475.90	2.5846	3	0.46
		model2. -475.19			
	LH	model1. -347.08	0.001	3	1
		model2. -347.08			
	CS	model1. -620.48	6.8873	3	0.075
		model2. -623.92			
***Staphylococcus succinus***	BRB	model1. -303.5	0.0014	3	1
		model2. -303.5			
	LH	model1. -279.39	0.0012	3	1
		model2.-279.39			
	CS	model1. -250.73	3.0948	3	0.37
		model2.-252.28			

**Table 2:** Summary of results from Cox proportion regression analysis of survivorship of sexually active and virgin males

**Pathogen**	**Host population**	**Mating status**	**Median time to death**	**% dead**	***p-value***
***Pseudomonas entomophila***	BRB	Mated	56.5	60	<0.0001
		Virgin	23.5	80	
	LH	Mated	…..	27	<0.0001
		Virgin	56.5	67	
	CS	Mated	21	90	<0.0001
		Virgin	18.5	100	
***Staphylococcus succinus***	BRB	Mated	…..	40	0.99
		Virgin	…..	35	
	LH	Mated	91	30	0.14
		Virgin	…..	40	
	CS	Mated	…..	40	0.31
		Virgin	.....	40	

**….** Mortality less than 50%.

To address this new finding in some depth, we used Pe and BRB males for our further studies.

### Resistance is at least partially responsible for higher survivorship

**Figure 3:**
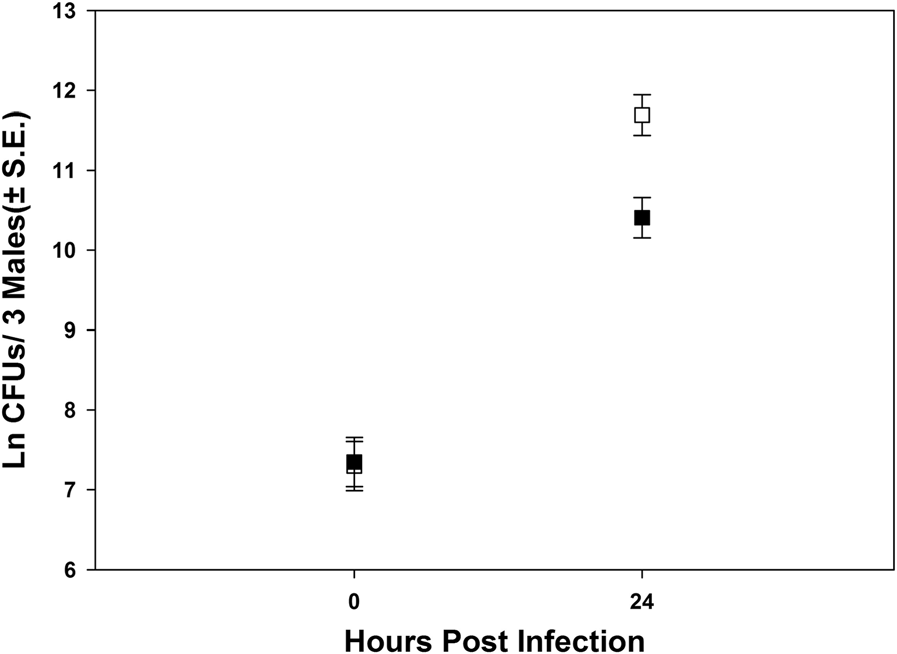
**Number of CFUs in sexually active and virgin males.** Virgin males (□) had significantly higher bacterial loads than sexually active males (■) 24 hours post infection (*p* = 0.02). However, the bacterial loads of virgin and mated males were not significantly different 0 hours after infection. Compared to the bacterial load at 0th hour post infection, the bacterial load of both virgin and mated males was higher at 24 hours post infection (*p* = 0.03), indicating that the bacteria grew within both virgin and infected males. Bacterial loads were determined by homogenizing flies in groups of three and plating them on LB agar plates which were then incubated at 27°C for 24 hours. The number of CFUs in each plate was then counted as a measure of bacterial load. The experiment had four independent blocks with 8–10 replicates within each block × treatment combination. The values plotted are Mean (± S.E.) averaged across the four block means. Data was analyzed using a three factor mixed model ANOVA where time and mating status were modeled as fixed factors crossed amongst themselves and with random blocks. The results are summarized in Table 3. Sham infected controls did not yield any colonies at 0 and 24 hours post infection (data not shown).

One potential reason for the observed difference in the survivorship of sexually active and virgin males upon infection could be differences in the bacterial growth rates within the two types of males i.e., differences in ‘Resistance’. To test this, we studied bacterial growth after 24 hours in infected males. We derived virgin and sexually active males as described before and infected them on 14th day post egg collection (i.e. 4th day post eclosion). We then estimated (a) the survivorship 24 hrs post infection and (b) the number of bacteria (Colony Forming Units- CFUs) within the flies at 0 and 24 hrs post infection. The whole experiment was repeated four times yielding 4 independent blocks. We used 8–10 plates per treatment × block combination to estimate CFUs (see Methods section for details). Consistent with the results from our previous experiment, we found that sexually active males survive significantly better than virgin males 24 hrs post infection (Mean survivorship ± S.E.; Virgins = 56 ± 1%; sexually active males = 72 ± 4%; paired *t* test: t = 4.51, df = 2, *p* = 0.04). Sexually active males produced significantly less number of colonies than virgin males (Figure 3). A three factor mixed model ANOVA treating mating status (virgin vs sexually active) and time (0 and 24 hrs post infection) as fixed factors and block as random factor indicated a significant effect of mating status, time and mating status × time interaction (Figure 3, Table 3). The number of CFU’s increased between 0 and 24 hrs indicating that the bacteria grew within both virgin and sexually active males (Figure 3, Table 3). Comparisons using Tukey’s HSD indicated that at 0 hours post infection, the bacterial load of virgin and sexually active males was not different while 24 hours post infection, sexually active males had significantly lower bacterial load compared to virgin males, thus leading to a significant interaction term (Table 3). Thus the growth of bacteria within the sexually active males was lesser than the bacterial growth in virgin males indicating that sexually active males probably have higher resistance to bacterial infection. In a matched control experiment, where flies were subjected to sham infection, none of the sham infected flies died. Additionally, sham infected flies did not yield any bacterial colony.

**Table 3:** **Effect of sexual activity on CFUs 24 hours after infection with *P.entomophila***

**Source**	**DF**	**DF Den**	**MS**	**F ratio**	***p*** **> F**
Mating status	1	82	9.82	5.58	0.02*
Hours	1	82	307.36	174.61	<0.0001*
Block & Random	3	82	67.72	38.46	<0.0001*
Hours × Block	3	82	13.12	7.45	0.00017^*^
Mating Status × Hours	1	82	8.41	4.78	0.03*
Mating Status × Block	3	82	0.73	0.41	0.74
Mating Status × Hours × Block	3	82	1.48	0.84	0.47

^*^Statistically significant values.

Summary of results from three-way mixed model ANOVA treating Mating status (virgin vs sexually active) and Hours (0 vs 24 hrs post infection) as fixed factors crossed with random blocks.

### Sexual activity is beneficial only if it precedes infection

**Figure 4:**
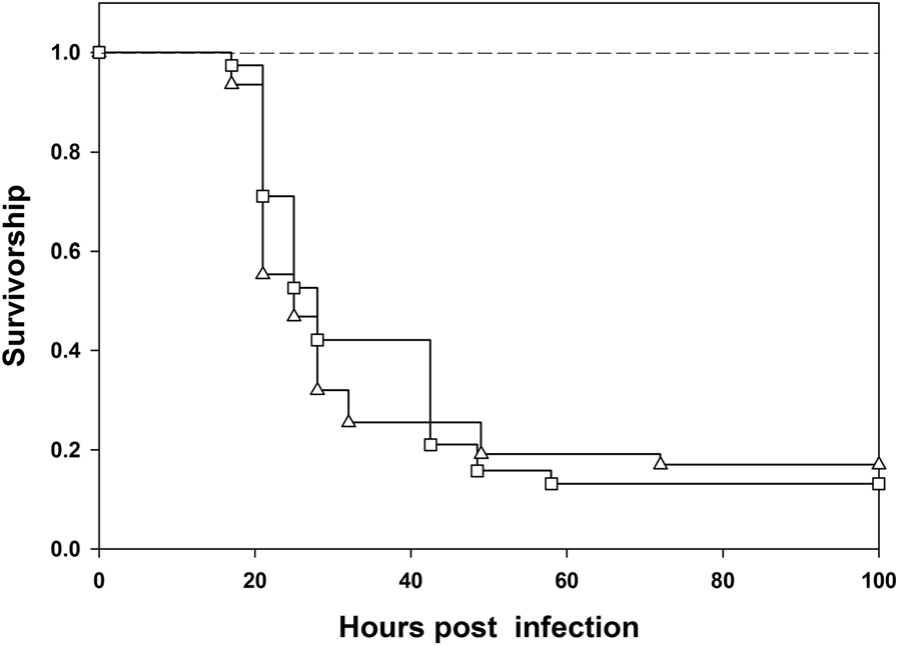
**Effect of sexual activity post infection on survivorship of males.** In this experiment, we infected virgin males (n = 100) with Pe. One hour after infection, 50 males were combined with virgin females while 50 were held in single sex groups. Mortality of the flies in the two treatments was then recorded. Survival of Virgin BRB males held with females post infection (Δ) was not different from that of Virgin males held without females (□) post infection (*p* = 0.76, Cox proportion regression analysis). None of the sham infected flies died. While we did have two types of sham infected controls (virgin males sham infected and virgin males sham infected and then combined with females), for simplicity, we represent both of them using a single line (―――) in the figure.

Virgin BRB males were collected on 10th day post egg collection. They were held as virgins for four days post eclosion in groups of five flies per vial. On 4th day post eclosion, virgin males were randomly assigned to two treatments: (1) Virgin males were infected with Pe and one hour after infection, were combined with virgin females (5 males and 5 females per vial, 10 vials) and (2) Virgin males were infected with Pe and continued to be held in single sex groups (5 flies per vial, 10 vials) post infection. When virgin males were first infected with Pe and then combined with virgin females, their survivorship was not significantly different from that of virgin males held without females post infection (Cox proportion regression analysis, *p* = 0.76) (Figure 4), indicating that the protective effect of sexual activity manifests only if it precedes infection. Ideally, in this experiment, we should have had a treatment where males were first held with females and then infected (similar to the sexually active male treatment described before). However, in multiple experiments, we observe that sexually active males have consistently better survivorship than virgin males. Hence, even though in this particular experiment we did not have a treatment where males had been sexually active prior to infection, we are confident that our conclusions are robust.

### Duration of male – female interaction affects survivorship

**Figure 5:**
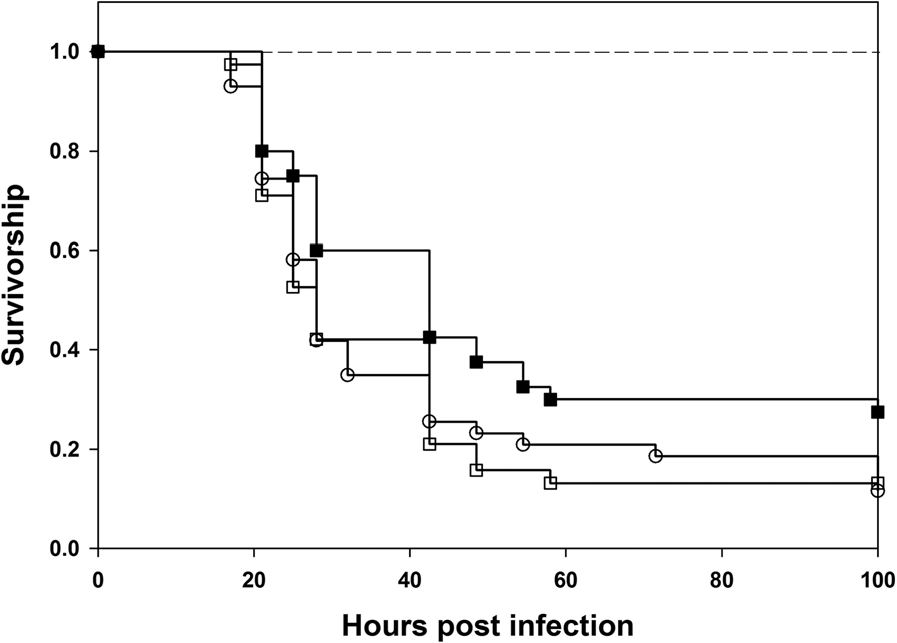
**Duration of male–female interaction affects male survivorship post infection.** Kaplan-Meier plots of survivorship of males differing in their duration of interaction with females. We generated three treatments- Males had no interaction with females and were held as virgins or Males and females interacted for one hour only or Males and females interacted for two days continuously (see section (f) in Methods for further details). The three types of males were then infected with Pe (n = 50 flies per treatment) and their survivorship post infection was monitored. Cox proportion regression was used to analyze data. Survivorship of Virgins (□) was not different from that of males held with females for one hour (○) (*p* = 0.99, Cox proportion regression). Males constantly held with females for two days (■) survived significantly better than both virgins (□) (*p* = 0.04, Cox proportion regression analysis) and males held with females for one hour (○) (*p* = 0.03, Cox proportion regression analysis). None of the sham infected controls died. Again, as in the previous figures, even though we had three different sham infected controls, we represent all of them using a single line (―――) in the figure.

We tested whether the duration of male–female interaction affected the survivorship of males post infection. Different treatments were created to alter the duration of male – female interaction. Two days post eclosion, virgin males were randomly divided into three groups: no interaction, short duration of interaction and long duration of interaction. For short duration of interaction, five 2-day old adult males were combined with five virgin females in a food vial (10 such vials were set up) for one hour and they were then separated. During this one hour, we observed mating take place in all the vials (data not shown). For long duration interaction, five 2-day old adult males were combined with 5 virgin females in a vial (10 such vials were set up) and held for two days after which, the males were infected. Virgins were held in single sex groups of 5 flies per vial (10 vials were set up) on all days. At the time of infection, all the males were 4-day old post eclosion. Males that interacted with females for only one hour did not differ from virgin males (Cox proportion regression analysis, *p* = 0.99) in terms of their survivorship, whereas, males held with females for two days (allowing for increased sexual activity/multiple matings) survived better than both virgin males and males allowed one hour of interaction time (Cox proportion regression analysis, *p* = 0.04 and 0.03 respectively) (Figure 5).

### Effect of intensity of sexual activity on survivorship

**Figure 6:**
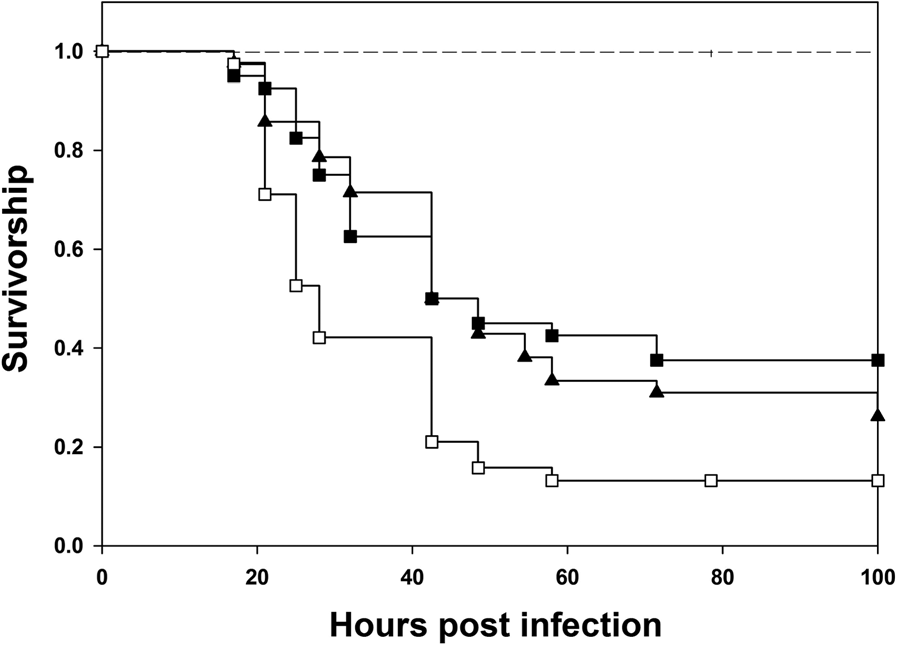
**Increased intensity of sexual activity does not affect male survivorship post infection.** Kaplan – Meier plots of survivorship of males varying in intensity of sexual activity. In the experiment virgin males were divided into three treatments varying in intensity of sexual activity: Males held as virgins and did not interact with females, Low interaction where one male was held with one female and High interaction where one male was held with four females. 50 flies per treatment were infected. Post infection survivorship of one male held with one female (▲) and one male held with four females (■) was significantly better than (□) Virgin males (*p* = 0.0017, Cox proportion regression). Survivorship of one male held with one female (▲) and one male held with four females (■) was comparable (*p* = 0.61, Cox proportion regression). Separate sham infected controls for all three treatments were run. No mortality in any of the sham infected controls was observed and thus, they are represented by a single line (―――) in the figure.

Previous studies [4,13] have suggested that the degree of sexual activity can affect male antibacterial immunity. In the present experiment, two day old virgin males were randomly assigned to one of the three treatments- (a) one male and one female per vial (b) one male and four females per vial and (c) five males per vial. Thus we created three treatments differing in the levels of sexual activity- no opportunity for males to interact with females (5 males in a vial, 10 vials), low opportunities for males to interact with females (one male and one female per vial, 50 vials) and high opportunities for males to interact with females (one male with four females per vial, 50 vials). Males in female biased vials are expected to have four times the amount of sexual activity than their equal sex ratio counterparts. After an exposure of two days, males were assayed for their survivorship post infection. Pair wise comparisons between treatments - (a) one male-one female and virgin males and (b) one male-four females and virgin males indicated significantly higher survivorship of sexually active males (*p* = 0.0091 and 0.0034 respectively, Cox proportional regression). However, we did not find any significant difference between the survivorship of males from the equal sex ratio and female biased sex ratio treatments (Cox proportion regression, *p* = 0.53) (Figure 6). Thus, unlike the previously reported results [5], there was no effect of increased level of sexual activity on survivorship of the experimental males.

### Protective effect of sexual activity decays over time

**Figure 7:**
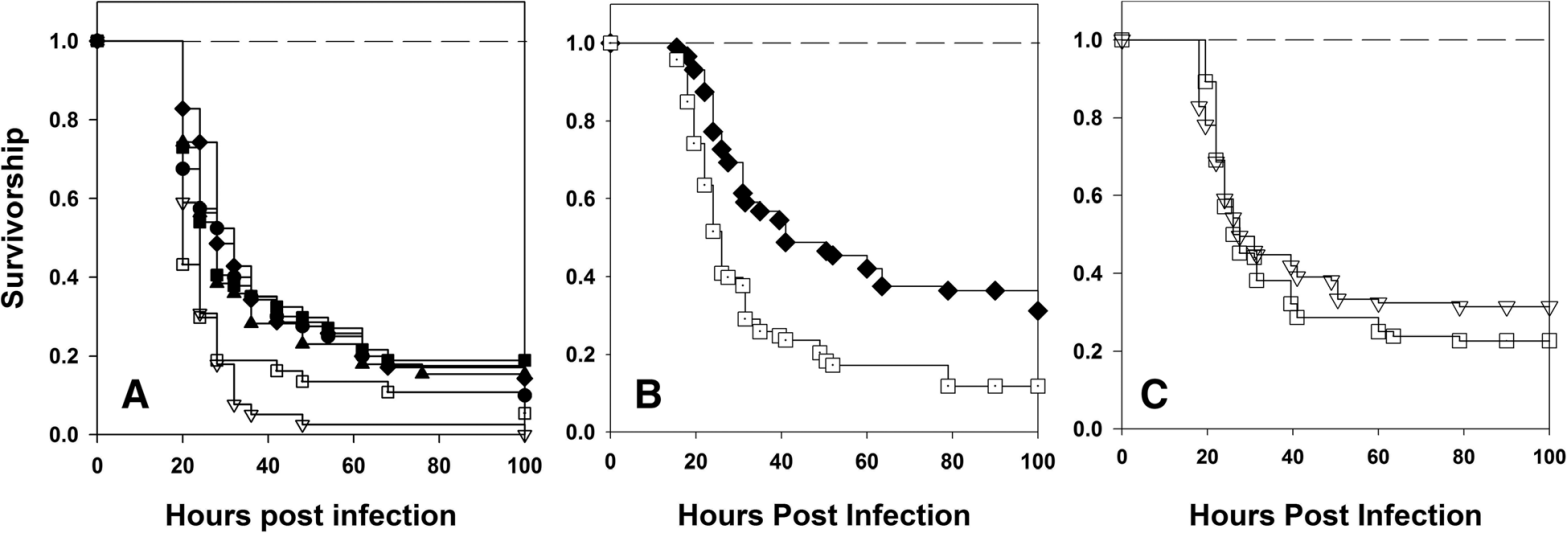
**Beneficial effects of sexual activity on male immunity declines over time.** The first experiment **(A)**, had six treatments: Virgin males (□), males allowed to interacted with females for two days and then separated from females and held in single sex groups for 4 (∇), 3 (▲), 2 (●), 1(♦) and 0 (■) days prior to infection (n = 50 per treatment). Males separated from females for 4 days had survivorship comparable to that of virgins (p = 0.51, Cox proportion regression). The survivorship of males separated from females for 3, 2, 1 and 0 days had significantly higher survivorship compared to both virgins and males separated from females for 4 days (all *p* < 0.05). The experiment was repeated in two more independent blocks with three treatments each- **(A)** Males separated from females for 4 days, **(B)** Males separated from females for 1 day and **(C)** a separate virgin male treatment for each of these two sexually active male treatments. Fifty males were infected for each treatment × block combination. We found no significant effect of block and hence pooled the data from the two blocks for analysis. Survivorship of males held as virgins (□) was significantly lower than survivorship of males separated from females for 1 day (♦) (**B**, *p* < 0.0001, Cox proportion regression analysis). Survivorship of virgin males (□) and males separated females for four days (∇) was not different (**C**, *p* = 0.4, Cox proportion regression analysis). Sham infected controls for all the treatments were run independently and no mortality was observed. All sham infected controls are indicated by a single dashed line.

To test whether the protective effects of sexual activity were time-dependent, we assayed the ability of sexually active males to survive an infection after denying them any further access to females for varying durations. Males were isolated as virgins on 10th day post egg collection and held in single sex groups of 5 per vial. The vials were then randomly assigned to six treatments. The males in the first treatment were held as virgins prior to infection. The males in the other five treatments were allowed to interact with females for two days, separated from females after the interaction period and then held without further access to females for (a) 4 days before infection, (b) 3 days before infection, (c) 2 days before infection, (d) 1 day before infection and (e) 0 days before infection. All males were of the same age at the time of infection and all infections were done on the same day (see Methods section for details). We used 50 males for each treatment. We observed that the positive effect of sexual activity on male survivorship decreased with the increase in the duration of mate deprivation (Figure 7A). Survivorship of males held without females for 4 days was not different from those of the virgins (Cox proportion regression analysis, *p* = 0.51). All other types of males had higher survivorship than virgin males (all *p* < 0.05).

We repeated this experiment in two more blocks where we had two treatments: 1. males separated from females for four days (after two day interaction with females) before infection and 2. Males separated from females for 1 day (after two day interaction with females) prior to infection. A separate virgin set was run with both the treatments. We used 50 males for each treatment × block combination. To check for an effect of block, same analysis using R packages coxme and coxphw was performed as mentioned before and no effect of block was observed (analysis of deviance, p = 1, df =3). Survivorship analysis showed that males which were separated from females one day prior to infection survived better than virgin males (Cox proportion regression analysis, *p* < 0.001) (Figure 7B) while the survivorship of males deprived of mates four days prior to infection was not significantly different from that of virgin males (Cox proportion regression analysis, *p* = 0.4) (Figure 7C), which is consistent with the result of the previous experiment.

Since survivorship of males held without females for four days and that of virgin males was not significantly different, we measured the bacterial load of flies from these two treatments 24 hours post - infection. We did not find any significant difference in the CFUs obtained from the two treatments (*t*-test, Mean ln CFU ± S.E.; Virgins = 9.3 ± 0.43; Males held without females = 9.5 ± 0.48; t = −0.34, df = 14, *p* = 0.75). Our earlier results (section b of Results) clearly show that sexually active males have lower bacterial loads compared to virgin males 24 hours post infection. Hence the present result, taken together with the previous result (on bacterial loads of sexually active and virgin males) indicates that the resistance of males increases with sexual activity but this increase in resistance is transient. When deprived of access to females for four days, resistance of males declines to the same levels as those of virgin males.

## Discussion

Our study clearly shows that sexual activity can have beneficial effects on immunity of males measured in terms of survivorship post infection in a pathogen dependent manner. When *Staphylococcus succinus* (Ss) was used as the pathogen, we found no difference between virgins and sexually active males in their post infection survivorship. However, when the pathogen was *Pseudomonas entomophila* (Pe), sexually active males had higher resistance compared to virgins as well as higher post infection survival rates. The protective effect of sexual activity on immunity was dependent on the duration of male–female cohabitation and the time gap between infection and the last interaction with females. This finding clearly challenges the widely accepted idea of a trade-off between sexual activity and immunity.

We observed an increase in resistance against Pe in sexually active males. The number of CFUs obtained from sexually active males 24 hrs post infection with Pe was significantly lower than those obtained from virgin males. However, the number of CFUs in virgins and mated males separated from females for four days was not significantly different indicating that the ability to resist Pe in mated males is a temporary effect which declines if further access to females is denied. These results clearly indicate that at least a part of the higher post infection survivorship (when infected with Pe) observed in sexually active males can be attributed to their increased resistance, i.e., ability to clear the pathogen and/or suppress the pathogens’ growth. We did not find any clear evidence of change in tolerance in sexually active or virgin males since bacterial load 24 hours post infection were different in these two treatments. Thus, it is most likely that the ability to resist Pe increases in sexually active males. We found that survivorship of virgin males and males held with females for one hour was not different from each other but males held with females for two days had significantly higher survivorship than either type of male. Thus, we find that the beneficial effects of sexual activity against Pe on survivorship post-infection are cumulative, being accrued over multiple male–female interactions. Increase in the intensity of sexual activity (male: female ratio of 1:4 compared to single mating pair per vial), however, does not alter the survivorship post infection, indicating a possible threshold beyond which increased sexual activity does not affect survivorship. Given the high mating rate in our host species, it is quite possible that this threshold is reached even when the treatment has a single mating pair. Therefore even though in female biased treatment (♂:♀ :: 1:4) mating rate is expected to be higher, it does not manifest in a difference in survivorship post infection. In previous studies using a gram negative pathogen *E. coli*, it has been shown that sexual activity and immunity (measured as bacterial load) are negatively correlated [5,14]. However, our study involving, a gram negative (Pe) and a gram positive (Ss) pathogen found no negative effect of sexual activity on male immunity. In *Tenebrio molitor*, singly mated males survive fungal infections better than virgin males [13]. Our results are in broad agreement with this study [13] in that mated males can survive bacterial infection better than virgin males.

There are at least two major reasons to explain the difference between our results and results of the other studies that did find a trade-off between immunity and sexual activity. First, immunity is a complex trait with multiple measureable components. In our study, we have measured survivorship post infection as an over-all measure of the health of the organism and its Darwinian fitness. Other studies that have found a tradeoff between sexual activity and immunity have assayed various functional components of immunity such as phenoloxidase activity, encapsulation, hemocyte load, hemolytic activity etc. (6–10). Thus, it is quite possible that in a given study, the presence or absence of trade-off between sexual activity and immunity depends on which components of immune system are assayed. Second, the trade-off between immunity and sexual activity seems to depend on the host-pathogen system being used in the study. In our own study, we found that the virgins and sexually active males survived equally well when the pathogen was Ss. However, when the pathogen was Pe, sexually active males had higher resistance and survived better than virgin males. Therefore, specific interactions between host and pathogen might determine the trade-off between sexual activity and immunity. The fact that we did not find any trade-off between immunity and sexual activity in males suggests that the proposed trade-off cannot be generalized. It is important to note that our study used three different populations of *Drosophila melanogaster* as hosts and two different pathogens. For each pathogen, we found consistent results across all three host populations, indicating that our results are robust across host populations.

The increased survivorship of sexually active males (compared to virgin males) upon infection with Pe is quite surprising and our results point towards probable underlying causes. Other studies [20] have found that if denied further access to females, the reproductive physiology of mated males progressively resembles that of virgin males over a three day period. Our finding that the protective effects of sexual activity decay over a three day period is suggestive of the involvement of changes in reproductive physiology in the increased resistance of the host [21]. However such changes offer protection against infections from Pe but not Ss indicating specificity in terms of protective effects of sexual activity.

## Conclusion

Our results suggest the necessity for caution while generalising the existing notion of an immunity-sexual activity trade-off in males. We show that sexual activity might increase host resistance against bacterial infection in a pathogen dependent manner. Further, the beneficial effects of sexual activity depend on the duration of male–female co-habitation and time from last mating. Our results indicate that immunity tradeoffs might be mediated through complex interactions between host, pathogen and the environment. Thus our results have major implications for the understanding of evolution of antibacterial immunity, reproductive behaviour and life-history related traits.

## Methods

### Host system (*Drosophila melanogaster)* and general fly handling

Two outbred, laboratory adapted populations – LH [20] and BRB and an inbred line (Canton S) were used as model hosts. BRB was established by mixing 18 wild-caught isofemale lines from Blue Ridge, USA and was maintained for 20 generations prior to the experiments. All the populations are maintained on 14 day discrete generation cycle, 12:12 LD regime, 25°C and 60-80% RH. The maintenance of LH populations has been described in detail previously [22]. LH is maintained on cornmeal-molasses food and BRB and Canton S on banana-jaggery food. For the experiments, flies were reared and maintained on their respective food.

In all our experiments, unless otherwise stated, flies were handled as follows: To generate flies for the experiments, eggs were collected from the stock populations and dispensed into food vials at a density of 100 eggs per vial. The vials were then incubated at 25°C temperature, 12:12 light –dark cycle at 50% RH. Flies were isolated as virgins (by isolating them within 6 hours of eclosion) from the peak of eclosion on the 10th day post egg collection. Flies were housed in single sex groups at a density of 5 flies per vial. At the time of infection, flies were typically four days old (post eclosion). For virgin treatments, males continued to be housed in single sex groups until infection. For treatments involving sexually active males, we combined 5 virgin males with 5 virgin females in a vial on 12th day post egg collection (ie 2-day post eclosion). The flies were allowed to interact for 2 days after which, the females were discarded and the males were infected. All males continued to be housed in single sex groups of 5 per vial post infection. Flies were transferred to fresh food vials every alternate day without anesthesia. Since we have used *Drosophila melanogaster* as host, no ethical approval was required.

#### Bacterial culture

Two pathogens gram negative *Pseudomonas entomophila* L48 [17] (Pe) and gram positive *Staphylococcus succinus subsp. succinus* (Ss) were used. *Pseudomonas entomophila* was isolated from the environment of *Drosophila melanogaster*[17] while *Staphylococcus succinus* was isolated from wild caught *Drosophila* [Singh K, Prasad NG: Unpublished data]*.* Protocol followed is reported previously in [23]. Briefly, bacterial culture was grown at 27°C and 37°C for Pe and Ss respectively till OD = 1 ± 0.1 following which cells were pellet down and suspended in equal volume of 10 mM MgSO_4_ before infection. Ss being a weak pathogen, the suspension was concentrated to OD 2.2 ± 0.1 before infection.

### Infection protocol and monitoring the flies for survivorship

Flies were lightly anesthetized using CO_2_. Flies were infected by pricking with a fine needle (*Minutein pin* 0.1 mm*,* Fine Science Tools, CA) dipped in bacterial suspension (bacteria suspended in 10 mM MgSO_4_) in the thorax. The control flies were pricked with a needle dipped in sterile 10 mM MgSO_4_ (sham infection). Flies were maintained at 25^0^C before, during and after infection with Pe and Ss. Flies were monitored for death post infection. We found no mortality over the first 18 hours post infection. Observations for mortality were made once every 4 hours from 18 to 60 hours post infection. After this period, observations were made in intervals of 6 hours (60 to 84 hrs post infection) and 8 hours (84 to 100 hrs post infection). This was followed for all the experiments.

Appropriate sham infected controls (i.e. virgin sham infected or sexually active sham infected as may be applicable) were run for all treatments. As there were no deaths observed in the controls in the observation window (i.e., till 100 hours post infection), all sham infected treatments in the figures are represented by a single dashed line for simplicity.

### Effect of sexual activity on male survivorship upon bacterial infection

Eggs were collected from the three different stock populations (BRB, LH and Canton-S) and flies were isolated as virgins as described before. The vials containing flies were randomly assigned to one of the two treatments- Sexually active and virgin. For the sexually active treatment, males and females were combined in food vials as described before on 12th day post egg collection and held for together for two days. The males in the virgin treatment continued to be held without females. On 14th day post egg collection, the males were infected. Half the males from the virgin treatment were infected with Pe and the other half with Ss. Similarly, half the flies from the sexually active treatment were infected with Pe and the other half with Ss. The flies were then observed for mortality. The experiment was done in two independent blocks (replicates). For each treatment × host population × pathogen × block combination, we infected 40 flies.

### Bacterial growth in flies

Flies were collected as virgins and on 12th day post egg collection (i.e. when the flies were 2-day old post eclosion), the vials were randomly assigned to one of the two treatments- Virgin or sexually active. Males in the sexually active treatment were combined with virgin females and were allowed to interact for two days as described before. Males in the virgin treatment continued to be held in single sex groups till the day of infection. One hundred Virgin and one hundred sexually active 4-day old (post eclosion), BRB males were infected with Pe. An equal number of flies were subjected to sham infection. After 0 and 24 hours post infection, males were homogenized in groups of three in 0.1 mL of 10 mM MgSO_4_**.** This homogenate was diluted (2 fold at 0 and 10 fold at 24 hrs) and 0.1 mL of the resulting suspension was plated on LB plates. Plating was done using WASP spiral plater (Don Whitley Scientific, UK) and plates were incubated at 27°C for 24 hrs. The resulting colonies were examined for their morphology to ensure similarity with Pe colonies. CFUs were counted using Acolyte colony counter (Don Whitley Scientific, UK). 8–10 such groups were used for each mating status × time × block combination. Experiment was done in four independent blocks. After 24 hours of infection, before taking out the flies for estimating bacterial load, we counted the number of dead flies in sexually active and virgin male treatments (in three blocks out of four) and subjected the data to a paired *t*-test. Plating of sham infected control flies that were pricked with sterile MgSO_4_ solution (as described above) yielded no bacterial colonies.

### Effect of sexual activity post infection

Flies were collected as virgins and held in single sex groups. Four days post eclosion, all the virgin males were infected. After one hour of infection, one set of 50 males was randomly chosen and combined with virgin females at a density of 5 males and 5 females per vial and 10 such vials were set up. Males and females were held together for the entire observation window (100 hours post infection). Virgin males continued to be held in single sex groups of 5 flies per vial. Ten such vials were set up for virgin treatment (hence n = 50).

### Duration of male female interaction

BRB males and females were collected as virgins and held in single sex group of five flies per vial. On 12th day post egg collection, males were divided into three treatments varying in the duration of interaction with females: (a) No interaction, (b) short duration interaction and (c) Long duration interaction. Males in the no interaction treatment were held as virgins (five per vial, 10 vials) throughout the experiment. For the short duration of interaction, males were combined with females at a density of five males and five females per vial (10 vials) for one hour. After one hour, females from the vials were removed using light CO_2_ and were discarded. Henceforth, males of short duration interaction were held in single sex vials of 5 males per vial. For the long duration interaction, five males and five females were combined in a vial (10 vials) and males and females were held together for two days till males were infected. On 14th day post egg collection males from the three treatments were infected with Pe. Males in all three treatments were held as single sex groups of five flies per vial after infection and there were ten such vials. Fifty flies per treatment were infected.

### Effect of altered sex ratios

BRB males were collected as virgins and held in single sex groups. On 12th day post egg collection, they were randomly assigned to one of the three treatments- (a) one male and one female, (b) one male and four females (c) five males. Using light CO_2_ anesthesia, males were combined with females at a density of one male and one female per vial (50 vials) or one male and four females per vial (50 vials). Virgin males (10 vials) were also anesthetized to account for possible effects of anesthesia. To control for variation in density (1 male:1 female vs 1 male: 4 female)), volume of the vial containing 1 male and 1 female was adjusted to one fifth by inserting cotton plug into the vial. Flies were held for two days followed by infection on 14th day post egg collection. The infected males were again held in groups of five flies/vial. Fifty flies were infected per treatment.

### Effect of time lapse between last interaction with females and occurrence of infection

Flies were collected as virgins and held in single sex groups. The vials were then randomly assigned to one of the six treatments. In the first treatment, males were held as virgins (5 flies per vial, 10 vials, n = 50) till the time of infection. In the second treatment, males were combined with virgin females without using anesthesia (5 pairs per vial, 10 vials, n = 50) on 12th day post egg collection. The males and females were allowed to interact for two days, after which the males were separated from the females (under light CO_2_ anesthesia) on 14th day post egg collection. Similarly, males in the third, fourth, fifth and sixth treatments were combined with females on 13th, 14th, 15th and 16th day post egg collection (5 pairs per vial, 10 vials per treatment, n = 50 males per treatment) and were separated from females on 15th, 16th, 17th and 18th day post egg collection. After the interaction time, the males were held in single sex groups of 5 flies per vial till the date of infection. Males from all the treatments were infected on 18th day post egg collection. Thus, flies from the different treatments had 4, 3, 2, 1 or 0 day gap between their last interaction with females and infection (while males in the virgin treatment had no interaction with females). Post infection, the males continued to be held at a density of 5 flies per vial. This experiment was repeated in two more blocks with each block containing three treatments - (a) virgin males, (b) males separated from females for one day (following a two day interaction period) before infection and (c) males separated from females for four days (following a two day interaction period) prior to infection. We ran separate virgin treatment with each of the mated treatment to avoid problems of multiple comparisons. Virgin males and females were combined in mating vials on 12th day and 15th day post egg collection. Males were allowed to interact with females for two days after which they were separated and again held in single sex groups of 5 flies per vial. All flies were infected on 18th day post egg collection. We infected 50 males for each block × treatment combination.

Bacterial growth was measured in two treatments: virgins and males separated from females four days prior to infection. Briefly, males were infected with Pe and 24 hours later they were crushed in groups of three in 0.1 mL of 10 mM MgSo_4_. The suspension was diluted 10 fold and 0.1 mL of it was plated. CFUs were recorded after 24 hours (see Methods section b and c for details). For each block × treatment combination, there were 8–10 such plates.

### Statistical analysis

Survivorship data were analyzed using Cox’s Proportional hazard model where death was recorded for each fly and flies not dead by the last time were treated as censored data. Analysis was implemented on JMP for windows, version 10 (SAS Inc 2012) and R (version 3.0.1) [24]. For assays done in two blocks, the data was modeled in two ways- (a) model1: that includes block as random factor (using R package ‘coxme’ [18]) and (b) model2: that does not include block as random factor (using R package ‘coxphw’ [19]). Analysis of deviance was performed comparing the log-likelihood ratio estimates of the two models. The log-likelihood ratios were not found to be different in any of the cases, thereby eliminating an effect of block (Table 1). Since there was no effect of block, data from both the blocks were pooled and the cumulative data was then tested for difference in survivorship. Survival curves were plotted using Kaplan – Meier Method (JMP for windows, version 10 (SAS Inc 2012)). To estimate the effect of mating status and time post infection on number of CFUs, colony count data was natural log transformed. Normality was verified using Shapiro – Wilk test. The data were then subjected to ANOVA treating mating status (sexually active vs virgin) and time post infection as fixed factors and block as a random factor.

To measure the effect of time elapsed between interaction with females and infection on number of CFUs, colony count data was natural log transformed and an unpaired t - test was performed.

## Competing interests

The authors declare that they have no competing interests.

## Authors' contributions

VG, ZSA and NGP designed the experiments. VG, ZSA performed the experiments. Analysis was done by VG, ZSA and NGP. Manuscript was written by VG, ZSA and NGP. All authors read and approved the final manuscript.
